# Distribution of hepatitis C virus genotype and subtype between Mongolian and Han in Inner Mongolia

**DOI:** 10.1097/MD.0000000000029545

**Published:** 2022-07-15

**Authors:** Ruijun Su, Li Dong, Yongxiang Wang, Renna Sa, Yafei Wang

**Affiliations:** Clinic Laboratory of the Affiliated Hospital of Inner Mongolia Medical University, Huimin district, Hohhot, P.R. China.

**Keywords:** hepatitis C, hepatitis C virus, genotypes distribution, prevalence, viral load

## Abstract

Hepatitis C is a serious infectious disease caused by the hepatitis C virus (HCV). HCV genotypes (GT) and subtypes are closely related to geographical distribution. Studies on the distribution of HCV genotypes can help to understand the regional epidemiology and genotype distribution and provide benefits in the treatment for hepatitis C.

To provide information about the distribution of HCV genotypes as well as improved prevention and treatment of hepatitis C, we aimed to classify the distribution of HCV genotypes among Mongolian and Han patients with hepatitis C in Inner Mongolia over the past 5 years.

Peripheral blood samples of patients with HCV were collected for gene sequencing. To analyze the HCV genotype distribution and possible influencing factors, we determined the viral load and ratios of various genotypes.

We found that the most prevalent genotype in Inner Mongolia was 1b, followed by GT2a, GT3a, GT3b, and GT6a. The prevalence of HCV among Mongolian patients was significantly higher than the prevalence in their Han counterparts (*χ*2 = 16.64, *P = *.000). There was no significant difference in viral load according to sex among HCV genotypes. However, the viral load of GT 1b was significantly higher than that of GT 2a (*F *= 3.51, *P = *.008). The viral load of GT 1b among ethnic Mongolians was significantly higher than that among Han patients (*t *= 2.28, *P = *.044).

The present study’s findings can serve as a basis for developing a personalized treatment for hepatitis C among patients in Inner Mongolia.

## 1. Introduction

Hepatitis C is a global epidemic caused by infection with the hepatitis C virus (HCV). HCV is a single stranded positive-sense RNA virus of the Flaviviridae family with diverse genetic variations. Accordingly, HCV is classified into 7 genotypes and at least 67 subtypes have been identified using the Simmonds genotype system.^[1]^ Different genotypes have distinct geographic distribution patterns related to the different epidemiological characteristics, including ethnic variability, in different regions. Infection with HCV affects people regardless of sex, age, or ethnicity. Furthermore, HCV is the main cause of chronic liver disease, which can lead to cirrhosis in approximately 20%–30% of patients after 20 to 30 years. Once cirrhosis occurs, 1%–4% of affected patients will develop hepatocellular carcinoma each year.^[2]^ According to statistics of the World Health Organization (WHO) in 2017, the prevalence of global HCV infection is about 3%; approximately 71 million persons have chronic hepatitis C worldwide, 10 million of whom are living in China.^[3]^ In 1990, Hepatitis C caused more than 225,000 deaths globally owing to cirrhosis, which increased to over 342,000 in 2017. Among these, 225,000 (65.8%) deaths in 2017 occurred in men and 117,000 (34.2%) in women.^[4]^ Furthermore, the mortality owing to liver cirrhosis and hepatocellular carcinoma caused by HCV infection accounts for approximately 25.5% of global deaths.^[4]^ As such, HCV infections are a serious public health problem worldwide.

The prevalence of HCV infection varies widely among different regions and countries. The total global prevalence of antiHCV + is estimated to be 1.6%, and the HCV RNA-positive prevalence is 1.1%.^[5]^ The prevalence of HCV ranges from 0.1% in certain Scandinavian countries to 23% in some African countries. However, 60% of the patients infected with HCV are in South and East Asia. According to the average prevalence (3.2%) of HCV, China has been considered a relatively high endemic area in terms of transmission route since 1993; however, measures have been implemented to slow HCV transmission over the past decades.^[6]^ The prevalence of HCV in the general population of China declined drastically from an intermediate prevalence (3.2%) in 1992 to a low HCV prevalence (1.3%) in 2014.^[2,5,7]^ Therefore, most provinces of China have become low-prevalence areas. The prevalence of HCV is geographically different throughout China, ranging from 0.32% to 6.51%. Most provinces with a lower prevalence are located in East and South China; Jiangxi and Guangdong provinces have a prevalence of 0.32% and 0.43%, respectively. Provinces with the highest HCV prevalence are Hubei and Liaoning, located in Central and Northeast China, with 6.51% and 2.88%, respectively.^[8]^ However, recent data regarding the HCV prevalence of Inner Mongolia are lacking.^[6,8,9]^

The global geographic distribution of HCV genotypes is variable and complex. Therefore, it is vital to investigate the epidemiological characteristics of HCV infection. Globally, genotype (GT) 1 is a common genotype with over one-third of the cases located in East Asia, accounting for 46% of more genotypes than any other single genotype, followed by GT3 (22%), 75% of which occurs in South Asia; GT2, GT4, and GT6 with 9.1%, 8.3%, and 5.4%, respectively. These genotypes are responsible for most cases of hepatitis C. In addition, the fewest HCV cases with GT5 occur in southern and eastern subSaharan Africa.^[5,6,10]^ GT7 was recently identified in an isolate from Central Africa.^[11]^ However, the prevalence of subtypes GT3a, GT3b, and GT6a has recently increased significantly, along with a decrease of subtypes GT1b and GT2a in East and Central China. The distributions of HCV subtypes differ throughout China. The main genotypes in Guangdong, Jiangxi, Guangxi, and Hunan provinces are GT1b and GT6a, whereas in Sichuan, Yunnan, and Chongqing the main genotypes are GT1b and GT3b.^[12]^ In Yunnan Province, GT6 is predominant (47%) followed by GT3 (41%) and GT1 (12%). HCV subtypes GT6n (30%) and GT3b (29%) are identified in 59% of intravenous drug users, experiencing a remarkable change over time.^[3,12,13]^

In addition to the Han population, the Inner Mongolia Autonomous Region has a large Mongolian population. According to the seventh national census, 3.4 million permanent residents are present in the Inner Mongolia Autonomous Region. However, data on the HCV genotype distribution among major ethnic minorities in this region are unavailable. We used quantitative detection and gene sequencing of HCV in peripheral blood samples of Han and Mongolian patients diagnosed with hepatitis C between 2013 and 2021. The current study was conducted to obtain a detailed overview of the HCV genotype and subtype distribution pattern among the different ethnic minorities in Inner Mongolia. We aimed to provide a clinical basis for the treatment of patients with HCV infection.

## 2. Materials and Methods

### 2.1. Patients

A total of 74,344 patients were tested for serum antiHCV at the Affiliated Hospital of Inner Mongolia Medical University from 2016 to 2021, including 39,034 male and 35,310 female patients. The results of the antiHCV test were exported from the database of the laboratory information system (LIS) of the hospital. The ethnicity, age, and sex of patients also were exported from the hospital LIS. Patients with repeated tests were excluded by comparing identifying information. We collected venous blood samples from all patients who tested positive for serum antiHCV in the LIS. We further obtained data of HCV RNA from the serum of corresponding patients. All experimental procedures were approved by the Medical Ethics Committee of Inner Mongolia Medical University (approval no. YKD2021253). All patients met the diagnostic criteria of the Guidelines for the Prevention and Treatment of Hepatitis C, which was formulated by the Chinese Society of Hepatology and Society of Infectious Diseases.

### 2.2. Laboratory tests

Tests for antiHCV and HCV RNA were performed at the Department of Clinical Laboratory of the Affiliated Hospital of Inner Mongolia University, which has been certificated by the China National Accreditation Service for Conformity Assessment (CNAS) since 2009 and in 2011, 2013, 2015, 2017, 2019, and 2021 passed the Laboratory Accreditation review. If a patient had several positive results for antiHCV, the first result was selected for analysis. Serum antiHCV testing was performed using an Abbott AXSYM i2000 (Abbott Laboratories, USA) and a chemiluminescence assay, according to the manufacturer’s instructions. An antiHCV level > 1.0 (ratio of optical density to cutoff [s/co]) was regarded as a positive result. To detect the HCV viral load, we used the Diagnostic Kit for Quantification of Hepatitis C Virus RNA (Da’an Gene Co., Ltd., Guangzhou, China), according to the manufacturer’s instructions. HCV genotype was performed on all samples that were positive for antiHCV using a Diagnostic Kit for Genotype of Hepatitis C Virus (PCR-reverse dot blot) (Da’an Gene Co., Ltd.), authorized by the China Food and Drug Administration. The lower detection limit of the diagnostic kit is 1000 copies/mL. The kit can detect HCV subtypes GT1b, Gt2a, GT3a, GT3b, and GT6a. Samples that cannot be detected using the diagnostic kit were subsequently analyzed using Sanger sequencing, as previously described.^[14]^ Two sets of universal primers targeting the core-envelope 1 (Core/E1) region of the HCV genome were used for nested PCR amplification and sequencing analysis (primer set 1: F1, 5′-GCAAC AGGGA ACCTT CCTGG TTGCT C-3′; R1, 5′-CGTAG GGGAC CAGTT CATCA TCAT-3′; primer set 2: F2, 5′-AACCT TCCTG GTTGC TCTTT CTCTA T-3′, R2, 5′-GTTCA TCATC ATATC CCATG CCAT-3′). The product lengths of primer set 1 and set 2 were 495 and 474 bp, respectively. The subtypes were obtained via phylogenic comparison to reference sequences for HCV using MEGA 4.0.^[15]^

### 2.3. Statistical analysis

The data were analyzed using IBM SPSS 21.0 software (IBM Corp., Armonk, NY) and presented as mean ± standard error of the mean, unless otherwise stated. The HCV prevalence rate and significant differences between HCV genotypes were performed using IBM SPSS 21.0 based on a chi-squared or Fisher exact test with Crosstabs in IBM SPSS. One-way analysis of variance and the least significant difference were used to analyze all experimental data of HCV viral load. Differences between the means of 2 groups were analyzed using an independent samples Student *t* test. *P* < .05 was considered to indicate a statistically significant difference.

## 3. Results

### 3.1. Characteristics of participants

A total of 74,344 patients were included in this investigation. Among these, 39,034 were men and 35,310 were women (ratio: 1.10:1); 71,610 were Han patients and 2734 were Mongolian. The patients’ average age was 51.8 ± 0.5 years.

### 3.2. Prevalence of hepatitis C in the patient population

In this study, the total positive rate of HCV antibody was 1.28% and that of HCV RNA was 0.83% (Table 1). Of 2734 Mongolian and 71,610 Han patients who were tested for serum antiHCV, 59 (2.16%) and 894 (1.25%) were positive for antiHCV, respectively. The rate of antiHCV positivity was significantly higher in Mongolian than in Han patients (χ2* *= 16.64, *P* = .000, OR = 1.74, 95% CI: 1.34–2.28); the same was true for HCV RNA (χ2* *= 9.18, *P* = .002, OR = 1.68, 95% CI: 1.20–2.34). Detailed information is given in Table 1.

**Table 1:** General information of Ethnicity and Sex populations with hepatitis C in Inner Mongolian, China.

Group		Ethnicity		Sex	
		Han	Mongolia	Male	Female
Total patients tested	Number, (%)	71,610 (96.3)	2734 (3.7)	39,034 (52.5)	35,310 (47.5)
	Age (mean ± SD)	52.0 ± 0.6	49.2 ± 2.0	50.6 ± 0.8	52.3 ± 0.8
	Total no. (%)	74,344 (100)		74,344 (100)	
AntiHCV positive	Number, (%)	894 (1.25)	59 (2.16)	535 (1.37)	418 (1.18)
	Age (mean ± SD)	51.8 ± 10.6	48.8 ± 15.2	50.8 ± 11.8	52.0 ± 11.6
	Total no. (%)	953 (1.28)		953 (1.28)	
HCV RNA >1000 copies/mL	Number (%)	581 (0.81)	37 (1.35)	349 (0.89)	269 (0.76)
	Age (Mean ± SD)	50.3 ± 12.7	50.1 ± 16.1	51.8 ± 14.2	52.8 ± 14.8
	Total no. (%)	618 (0.83)		618 (0.83)	

Of the 39,034 male and 35,310 female patients tested for HCV antibody, 535 (1.37%) and 418 (1.18%) were positive for HCV antibody, respectively. The rate of HCV antibody positivity was significantly higher in men than that in women (χ2* *= 4.98, *P* = .025, OR = 1.16, 95% CI: 1.02–1.32); the same was true for HCV RNA (χ2* *= 3.87, *P* = .049, OR = 1.18, 95% CI: 1.00–1.38). Detailed information is shown in Table 1.

### 3.3. Distribution of HCV genotypes and subtypes

**Figure 1.**
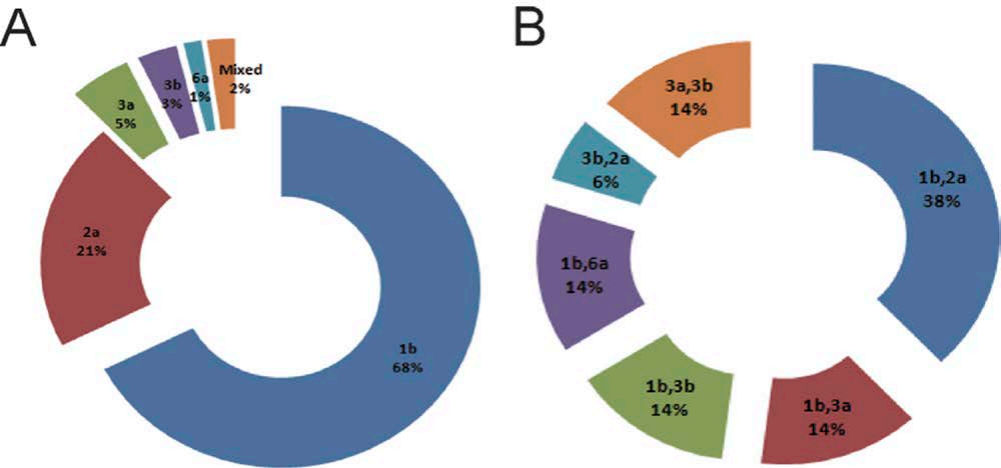
Distribution of hepatitis C virus (HCV) genotypes among patients in Inner Mongolia. (A) Constituent ratio (%) of HCV subgenotypes among patients. (B) Constituent ratio (%) of HCV subgenotypes among patients with mixed infection.

Among the 953 patients who were positive for antiHCV, 618 were successfully genotyped. We detected 5 genotypes among these patients: GT1b, GT2a, GT3a, GT3b, GT6a, and GT1b, accounting for 68.0%; we also detected GT2a (21.0%), GT3a (5.0%), GT3b (3.0%), and GT6a (1.0%). There were 2.0% cases of mixed infection with 2 genotypes or subtypes identified. Among cases of mixed infection, the combinations of HCV genotype/subtype were 1b/2a (38.0%), GT1b/3a (14.0%), GT1b/3b (14.0%), GT1b/6a (14.0%), GT3b/2a (6.0%), and GT3a/3b (14.0%). Mixed infections with 3 subtypes were not observed in our study. Detailed information is presented in Fig. 1.

The distribution of genotypes among Han and Mongolian patients showed no significant differences (Table 2). Because of the limited sample size, GT6a was not found in Mongolian patients in our study. However, the rate of GT2a among women was significantly higher than that in men (χ2* *= 6.52, *P* = .011, OR = 0.60, 95% CI: 0.41–0.89). Moreover, there was a higher proportion of GT3a and GT3b identified among men than women (χ2* *= 12.45, *P* = .001, OR = 5.55, 95% CI: 1.92–16.1; χ2* *= 6.84, *P* = .008, OR = 4.54, 95% CI: 1.32–15.7). However, the difference in GT1b between men and women was not significant, according to Fisher exact test (χ2* *= 0.36, *P* = .545, OR = 0.90, 95% CI: 0.64–1.26). Details are shown in Table 3.

**Table 2:** Constituent ratio (%) of hepatitis C virus (HCV) genotypes between Han and Mongolian patients in Inner Mongolia.

HCV genotypes	Han		Mongolian		χ2	*P* value	OR (95% CI)
	Number	Ratio	Number	Ratio			
1b	394	67.8	23	62.2	0.506	0.476	1.14 (0.57–2.26)
2a	117	20.1	10	27.0	1.011	0.315	0.68(0.32-1.45)
3a	30	5.2	1	2.7	0.076	0.783	1.96(0.26-14.8)
3b	18	3.1	2	5.4	0.084	0.772	0.56 (0.12–2.50)
6a	9	1.5	0	0.0	—	—	—
Mixed	13	2.2	1	2.7	0.149	0.699	0.82 (0.10–6.48)

**Table 3:** Distribution of hepatitis C virus (HCV) genotypes in men and women (cases) in Inner Mongolia.

HCV genotypes	Men (n = 349)	Women (n = 269)	χ2	*P* value	OR (95% CI)
1b	232 (66.5%)	185 (68.8%)	0.365	0.545	0.90 (0.64–1.26)
2a	59 (16.9%)	68 (25.3%)	6.524	0.011	0.60 (0.41–0.89)
3a	27 (7.7%)	4 (1.5%)	12.452	0.001	5.55 (1.92–16.1)
3b	17 (4.9%)	3 (1.1%)	6.843	0.008	4.54 (1.32–15.7)
6a	7 (2.0%)	2 (0.7%)	0.922	0.337	2.73 (0.56–13.3)
Mixed	7 (2.0%)	7 (2.6%)	0.244	0.621	0.77 (0.26–2.21)

### 3.4. HCV genotypes/subtypes and viral loads

**Figure 2.**
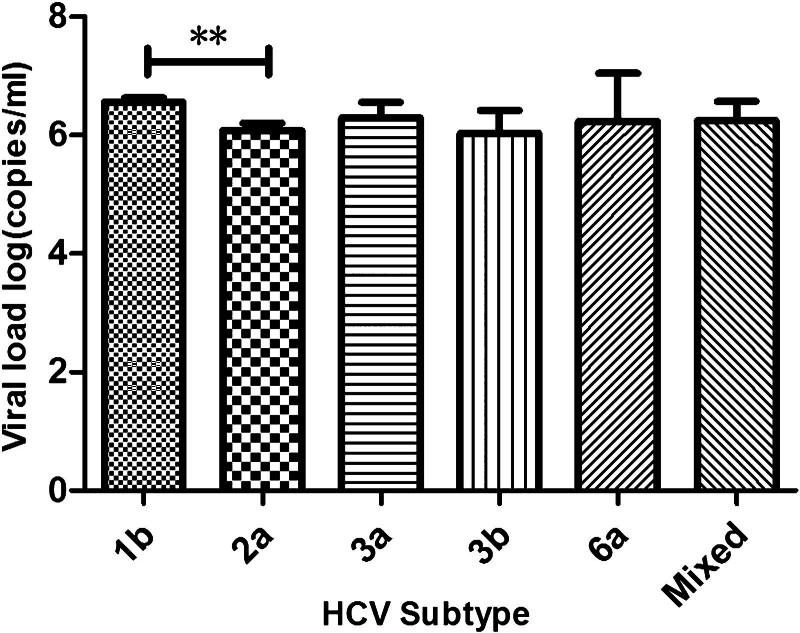
Viral load of hepatitis C virus (HCV) subtypes among patients in Inner Mongolia. Data are presented as mean + standard error of the mean. One-way analysis of variance and least significant difference were used to analyze data of HCV viral load (*F *= 3.51, *P = *.008). **P < .01 genotype (GT) 1b vs GT2a.

HCV genotypes/subtypes in the viral load results were obtained for 618 samples. We analyzed the relationship between subtype and viral load. The log of average viral loads among subtypes was 6.56 ± 0.07, 6.07 ± 0.13, 6.29 ± 0.26, 6.04 ± 0.38, 6.24 ± 0.81, and 6.25 ± 0.32 for GT1b, GT2a, GT3a, GT3b, GT6a, and mixed genotype, respectively (Fig. 2). The log of average results was significantly different among the subtypes (*F *= 3.51, *P = *.008). In addition, the log of average viral load was higher in subtype GT1b than in GT2a (*P* < .01).

**Figure 3.**
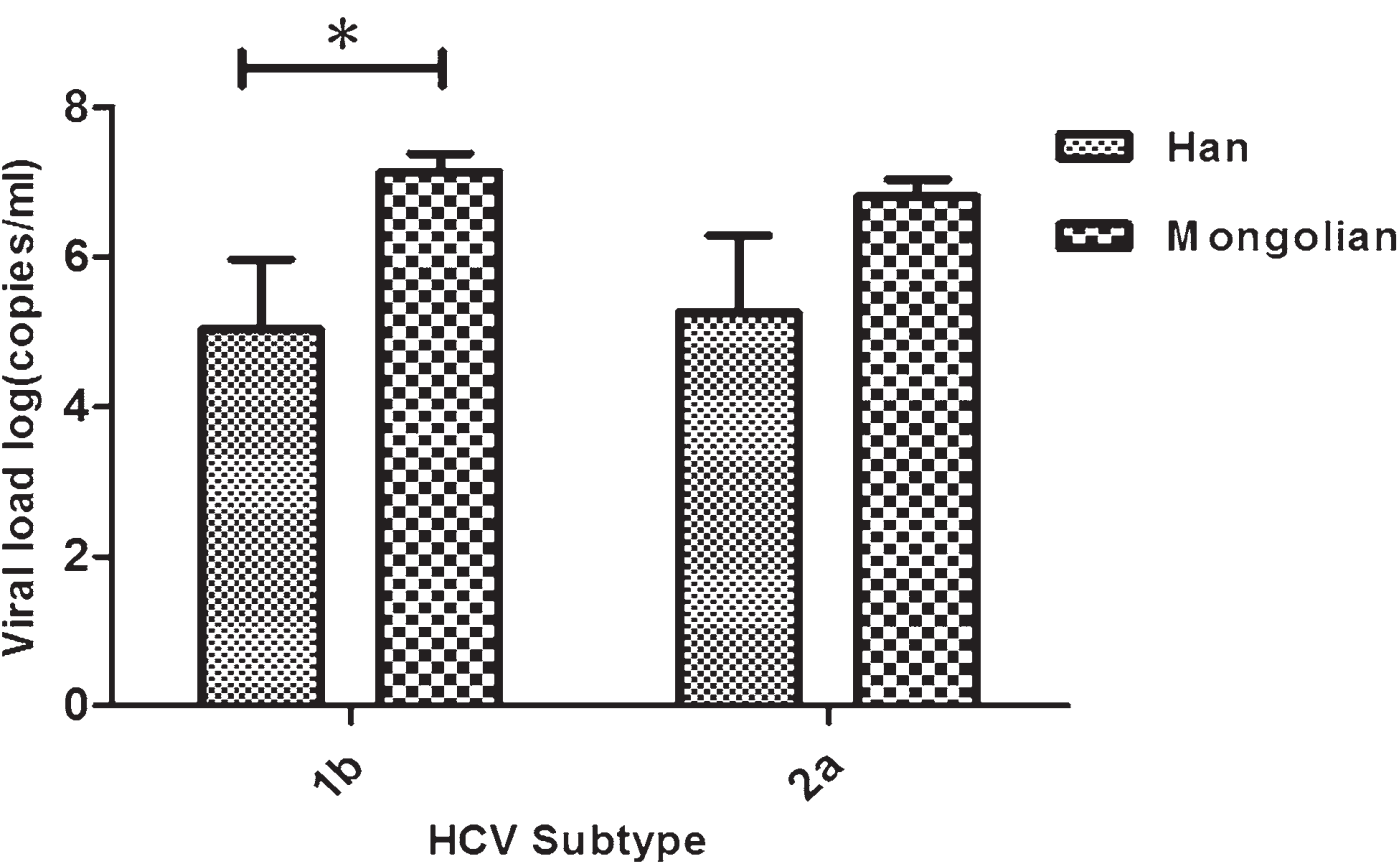
Viral load of hepatitis C virus (HCV) subtypes between Han and Mongolian patients in Inner Mongolia. Viral load of minor subtypes (genotype [GT]3a, GT3b, GT6a, Mixed) were not compared because of a lack of samples. Data are presented as mean + standard error of the mean. Differences between the means of 2 groups were analyzed using an independent samples Student *t*-test. *P < .05 Han vs Mongolian in GT1b group (GT1b: *t *= 2.28, *P = *.044; GT2a: *t *= 1.54, *P = *.126).

The log of the average viral load of GT1b was higher in Mongolian than in Han patients (*P* = .044). Although it did not reach statistical significance (*P = *.126), the log of average viral load in the GT2a group of Mongolian patients showed a higher trend than that of Han patients. Fig. 3 shows these results.

**Figure 4.**
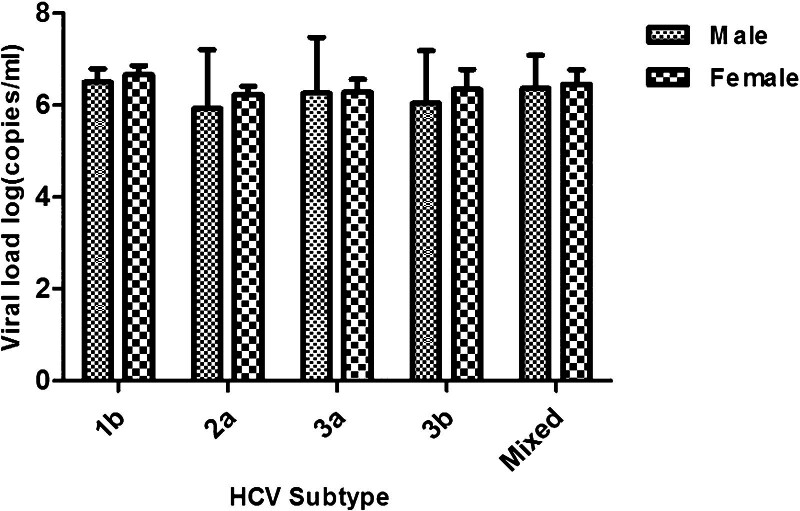
Hepatitis C virus (HCV) subtype viral load in male and female patients in Inner Mongolia. Viral load of minor subtype (genotype 6a) was not compared because of a lack of samples. Differences between the means of 2 groups were analyzed using an independent samples Student *t*-test. Data are presented as mean + standard error of the mean.

Meanwhile, the log of average viral load in all the subtypes group of Women showed a higher trend than men. However, there were no significant differences in the viral load level between men and women (Fig. 4).

## 4. Discussion

With the successful use of direct antiviral drugs, chronic hepatitis C has become a clinically curable disease. These drugs offer the potential for HCV eradication. However, effective screening and appropriate timing of treatment in patients infected with HCV play key roles in the prevalence of HCV. It is very important for appropriate and accurate screening methods during this process.^[16,17]^ Patients infected with HCV are identified via testing for serum antiHCV and HCV RNA. Serum antiHCV detection is a sensitive and convenient method for screening patients infected with HCV whereas detection of HCV RNA is considered the “gold standard” for the clinical diagnosis of patients with HCV infection. HCV viral load is the main criterion for assessing the efficacy of antivirus medications.^[18]^ In this study, serological antibody tests were used to screen the general population and HCV RNA testing was used to confirm individuals with HCV infection.

The Affiliated Hospital of Inner Mongolia Medical University includes all clinical departments and the Physical Examination Center and has a large number of patients. Most patients reside in the Inner Mongolia Autonomous Region. It has 3120 beds and receives approximately 2128,000 outpatients every year. It is noted for its advantages in the treatment of infectious diseases, trauma, and acute and severe diseases. Although our data were obtained from the LIS system of a single institute, our sample can provide a good representation of the prevalence of HCV in the Inner Mongolia Autonomous Region. In the present study, we investigated the prevalence of HCV genotypes and subtypes in Inner Mongolia and confirmed the circulation of 4 HCV genotypes (1, 2, 3, and 6) and 5 HCV subtypes (1b, 2a, 3a, 3b, 6a) (Fig. 1A). Overall, the antiHCV positivity rate was 1.28% in this study (Table 1), which is higher than the rate of 0.93% reported previously in China.^[8]^ It can be seen that hepatitis C in Inner Mongolia has a high incidence compared with the rest of the country. In addition, the rate of antiHCV positivity was significantly higher in Mongolian patients than in Han patients in our study; the same was true for HCV RNA (Table 1). In the incidence of hepatitis C, it is closely related to ethnicity, among which being Mongolian is a high-risk factor. Although the precise reasons for this difference in the prevalence of antiHCV according to ethnicity are not yet clear, it may be related to different lifestyles. Mongolian individuals may have a greater likelihood of being infected with HCV.^[19,20]^ Another possibility is closely related to the genetic background and autoimmune status of HCV-infected individuals.^[21,22]^ Single nucleotide polymorphism in the human leukocyte antigen alleles may play an important role in immune-mediated diseases, including liver cancer.^[23]^ We will focus on these points in future research.

Globally, GT1b is the most common subtype, accounting for 22% of all genotypes. Subtypes GT1a and GT1b, with a ratio of 1:2, account for 90% of all GT1 strains.^[6]^ A recent meta-analysis showed that GT1b and GT2a, accounting for 62% and 17%, respectively, are the predominant HCV genotypes in China. Subtypes GT1b and GT2a comprise the greatest proportion of HCV in China. Our study revealed 5 HCV subtypes (1b, 2a, 3a, 3b, 6a) are prevalent in the Inner Mongolia Autonomous Region; others published studies have shown similar results.^[12,14]^ It has been reported that GT1b (63%) and GT2a (33%) are the predominant subtypes in Inner Mongolia, followed by GT3a and GT1a.^[12]^ In this study, GT1b and GT2a were the main subtypes in the Inner Mongolia Autonomous Region, but we did not detect GT1a (Fig. 1A). In recent years, the proportion of patients with coinfection by multiple subtypes has shown an upward trend. These subtype coinfections involve GT1b, GT2a, and GT6a, among which GT1b/2a coinfection is the most common, accounting for 81.8%. GT1b/2b and GT2a/2b coinfections are only occasionally reported in China.^[12]^ In this study, most combinations of HCV genotype/subtype in coinfections were GT1b/2a (38.0%), followed by GT1b/3a (14.0%), GT1b/3b (14.0%), GT1b/6a (14.0%), GT3b/2a (6.0%), and GT3a/3b (14.0%) (Fig. 1B). Comparing the results of our study with those of other studies in China, we found that the HCV genotype and subtype distribution pattern of GT1b/2a represented the most common coinfection. In contrast, coinfections with GT3b/2a and GT3a/3b were common in Inner Mongolia Autonomous Region. Coinfection with 3 subtypes was not observed in our study. However, it has been reported that a high percentage of triple infections of HCV is present in Gansu Province, of which GT1a/1b/2a or GT1a/1b/2c were more common.^[12,24]^ Furthermore, patients who repeatedly receive blood transfusion or routine dialysis well as drug users who share needles significantly contribute to multiple HCV genotype/subtype coinfections in China.^[12,25,26]^

Sex differences are reported in antiHCV-positive rates. Positive rates of both serum antiHCV and HCV RNA in male patients with hepatitis C are significantly higher than those in female patients, according to recent studies.^[3,27,28]^ The results of our study demonstrated that the prevalence and distribution of HCV genotypes varied with sex, with a significantly higher prevalence of HCV infection in men than in women (Table 1). The prevalence of HCV is closely related to sex, among which being male is a high-risk factor. Sex is an important risk factor for the distribution of HCV genotypes. Although the precise reasons for this difference are unclear, environmental exposures and lifestyle, such as male homosexuality, sharing of needles used for drug injection, receiving tattoos, and alcohol consumption, may make men more likely to be infected with HCV.^[29]^ In addition, some studies suggest that women have higher rates of viral clearance and a better response to HCV treatment than men.^[30]^ HCV subtypes GT3a and GT3b are more prevalent in men than in women. However, the distribution of subtype GT2a in male carriers was much lower than that among women in this study (Table 3). The different distribution of HCV subtypes according to sex was likely the result of a biological mechanism in HCV infection. The exact mechanism is unknown, but estrogen has been revealed as a cause in 1 hypothesis.^[27]^ In our study, no difference in relation to sex was observed among the subtype groups of HCV RNA level (Fig. 4). Sex was not a significant risk factor for subtype groups of viral load in the present study. Furthermore, some studies have indicated that women tend to have a higher rate of viral clearance.^[31]^ Hence, more data are needed to ascertain the risk factors and mechanisms involved in this process.

We found that the prevalence and distribution of HCV genotypes varied with ethnicity, although the distribution of subtypes was not significantly different between Han and Mongolian patients (Table 2). We also found that the viral load of GT1b was significantly higher in Mongolian than patients those in Han. That means GT1b has a higher viral titer in Mongolian patients. Although there was no difference between Han and Mongolian patients, the average viral load in the GT2a group of Mongolian patients showed a higher trend than that of Han patients (Fig. 3). Some studies suggest that GT1b is a major risk factor for developing hepatocellular carcinoma compared with other subtypes.^[2]^ This conclusion can also be inferred from our results. In this study, the log of average viral load was higher in subtype GT1b than that of GT2a in all patients (Fig. 2). Because patients infected with GT1b have a lower possibility of sustained virological response, they are recommended to undergo a longer duration of therapy. The higher incidence of hepatocellular carcinoma possibly resulted from the lower response rate of patients infected with GT1b. Moreover, the findings suggested that the genotype of HCV infection had an impact on the prognosis in advanced clinical stages.^[2,32]^ Therefore, Mongolian patients infected with GT1b may have a more serious disease than Han patients.

## 5. Conclusions

Our study suggested that there are 5 HCV subtypes present in Inner Mongolia Autonomous Region, GT1b, GT2a, GT3a, GT3b, and GT6a, with subtype GT1b predominating, followed by GT2a and GT3b. HCV prevalence and genotype distribution were found to have a certain relationship with sex and ethnicity. There was a high incidence of HCV in Inner Mongolia compared with the rest of China. The incidence of hepatitis C in the Mongolian population was significantly higher than that in the Han population, and the disease may be more severe in Mongolian patients. To our knowledge, this is a simple report to evaluate the prevalence and distribution of HCV genotypes in Inner Mongolia, so it has some limitations, such as a relatively small sample size and possible selection bias with the use of hospital data. However, our study findings may help understand the genotype distribution of HCV in Inner Mongolia. Our findings can also provide important information about HCV prevalence according to ethnicity and sex. Estimates of HCV prevalence by ethnicity and sex can help health authorities to understand their local epidemic and develop targeted prevention and treatment programs. Utilizing these population-based results with a local understanding of HCV risk factors and treatment practices can provide greater insight into the hepatitis C epidemic in China and globally.

## Acknowledgments

We thank LetPub (www.letpub.com) for its linguistic assistance during the preparation of this manuscript.

## Author contributions

Ruijun Su and Dong Li designed the study; Rujun Su and Yongxiang Wang performed the research; Renna Sa analyzed the data; and Rujun Su wrote the manuscript. Yafei Wang gathered the data; Dong Li helped perform the analysis with constructive discussions. All authors read and approved the final manuscript.
